# Biocompatible organic–inorganic hybrid materials based on nucleobases and titanium developed by molecular layer deposition

**DOI:** 10.3762/bjnano.10.39

**Published:** 2019-02-08

**Authors:** Leva Momtazi, Henrik H Sønsteby, Ola Nilsen

**Affiliations:** 1 Centre for Materials Science and Nanotechnology (SMN), Department of Chemistry, University of Oslo, P.O. Box 1033 Blindern, N-0315 Oslo, Norway

**Keywords:** ALD, bioactive materials, hybrid materials, MLD, nucleobases

## Abstract

We have constructed thin films of organic–inorganic hybrid character by combining titanium tetra-isopropoxide (TTIP) and the nucleobases thymine, uracil or adenine using the molecular layer deposition (MLD) approach. Such materials have potential as bioactive coatings, and the bioactivity of these films is described in our recent work [Momtazi, L.; Dartt, D. A.; Nilsen, O.; Eidet, J. R. *J. Biomed. Mater. Res., Part A* **2018,** *106,* 3090–3098. doi:10.1002/jbm.a.36499]. The growth was followed by in situ quartz crystal microbalance (QCM) measurements and all systems exhibited atomic layer deposition (ALD) type of growth. The adenine system has an ALD temperature window between 250 and 300 °C, while an overall reduction in growth rate with increasing temperature was observed for the uracil and thymine systems. The bonding modes of the films have been further characterized by Fourier transform infrared spectroscopy, X-ray photoelectron spectroscopy and X-ray diffraction, confirming the hybrid nature of the as-deposited films with an amorphous structure where partial inclusion of the TTIP molecule occurs during growth. The films are highly hydrophilic, while the nucleobases do leach in water providing an amorphous structure mainly of TiO_2_ with reduced density and index of refraction.

## Introduction

There is an ever-increasing interest in organometallic compounds in the field of medicinal chemistry. Organometallic complexes are now being developed as anticancer agents, radiopharmaceuticals for diagnosis and therapy, and probes for biosensing [1]. Nucleobases are constituents of DNA and RNA and can interact with different metals to form several molecular assemblies [2–3]. In the 1960s, a powerful antitumor agent named cisplatin (*cis*-[Pt(NH_3_)_2_Cl_2_]) was discovered by Rosenberg [4]. Later it was realized that the mode of action of this drug is due to coordinative bond formation of the metal ion with nucleobase donor atoms in DNA. This led to an upsurge in interest in organometallic compounds containing nucleobases [5–7]. For instance, attempts have been made to bind adenine to dirhodium anticancer complexes for better metallo-pharmaceutical activity and toxicity reduction [8].

This work describes the growth of films based on such organic nucleotides as complexes with the biocompatible metal titanium. As thin films, such materials can be better applied as coatings on scaffolds or implants where interaction with the surrounding tissue is controlled at the surface of the material, whereas load is governed by bulk properties. The surface of a material is responsible for interactions with the surrounding tissue by directing protein absorption, which in turn controls cell adhesion and response [9]. Thus, the tailoring of the surface of materials used in tissue engineering is important for designing bioactive and biocompatible materials.

Our choice is the atomic layer deposition/molecular layer deposition (ALD/MLD) technique by which organic–inorganic materials are developed through the gas phase. ALD was initially developed for production of thin films of zinc sulphide [10], and since its introduction in the 1970s, it has expanded to include a wide range of materials, including organic–inorganic hybrid compounds. In both ALD and MLD, the precursors are introduced onto the surface of a substrate sequentially, separated by purging steps to remove byproducts and unreacted precursors. The reactions occur ideally in a self-limiting, surface-saturated manner [11–13], which ensures uniform coverage even on complex geometries. Molecular-level control of the deposited film can be achieved due to the cyclic nature of the process and enables variation of the type of building units as the film grows [14]. MLD is a special case of ALD where larger molecular fragments, such as nucleobases, are used as building blocks for film growth [15–16]. The design of a film at the molecular level enables control of cell–surface interactions, which plays a major role in controlling the bioactivity of solid surfaces.

Biocompatibility can be enhanced by coating the surface using various thin film deposition techniques such as chemical vapor deposition (CVD), physical vapor deposition (PVD) or atomic layer deposition (ALD) [17]. A coating produced by the ALD/MLD methods can render a non-biocompatible surface of an implant into a biocompatible material, allowing for the use of alternative materials as implants [18]. Moreover, ALD can provide specialized surface functionalities useful for biological applications [19]. Such biocompatible surfaces have not been widely adapted within use of ALD, although recent attempts have been reported for deposition of biocompatible hydroxyapatite thin films [20] and hydrophilic ALD-deposited alumina thin films [21], in addition to our prior work on titaminates [22], and our recent investigation of biocompatibility of a selection of ALD materials, including the films developed here [23]. Despite these attempts, the design of biocompatible and bioactive surfaces is a huge field where ALD/MLD has only just begun to provide solutions.

**Figure 1:**
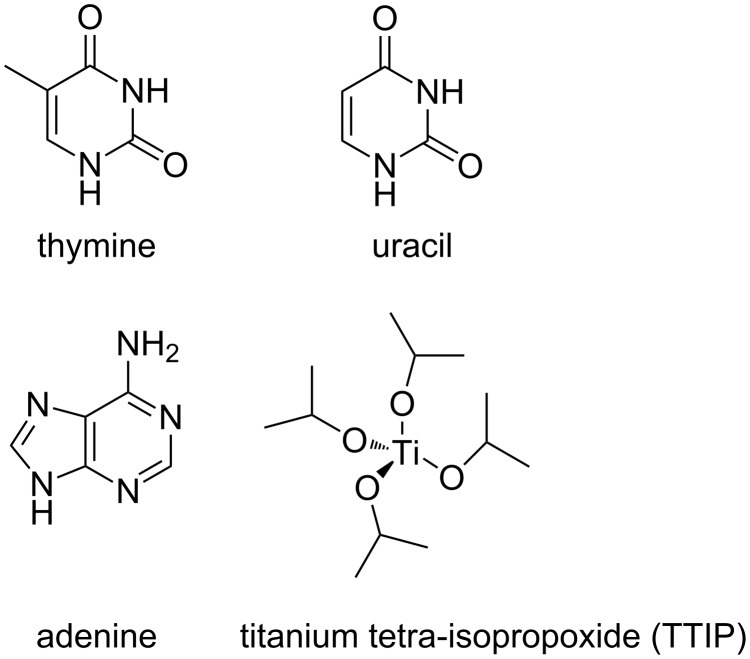
The organic and inorganic molecules used as precursors in the current study.

The current work is based on our previous study on organic–inorganic MLD materials, in which we used amino acids as organic linkers to form titaminates [22]. We here build on that knowledge and expand the range of bioactive MLD materials to now include nucleobases as organic linkers. The films have been prepared by combining titanium tetra-isopropoxide (TTIP) with thymine, uracil, and adenine (Figure 1). We have recently reported a significant increase in the proliferation rate of rat conjunctival goblet cells cultured on substrates coated with these hybrid materials based on amino acids, and also the currently presented nucleobases, compared to uncoated glass coverslips using alamarBlue^®^ proliferation assay [23]. The current contribution describes the growth of the films based on nucleobases in more detail.

## Results

**Figure 2:**
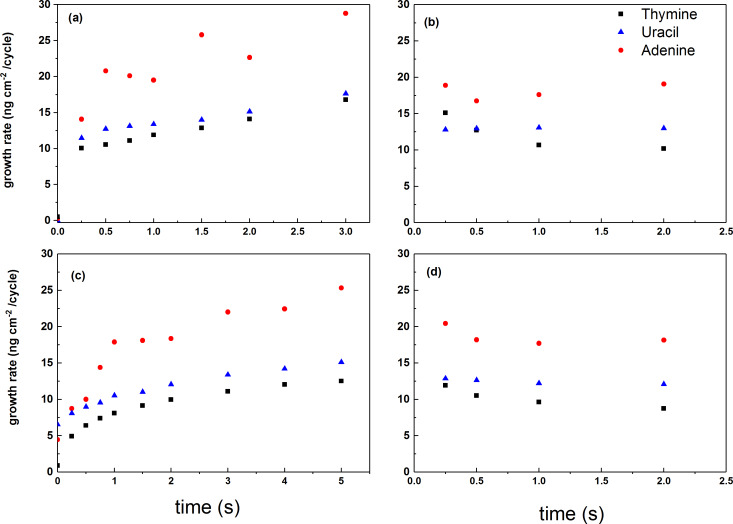
The growth rate of the QCM systems as function of (a) length of TTIP pulse, (b) TTIP purge, (c) organic precursor pulse (thymine – black square, uracil – blue triangle, adenine – red circles), and (d) organic precursor purge.

**Figure 3:**
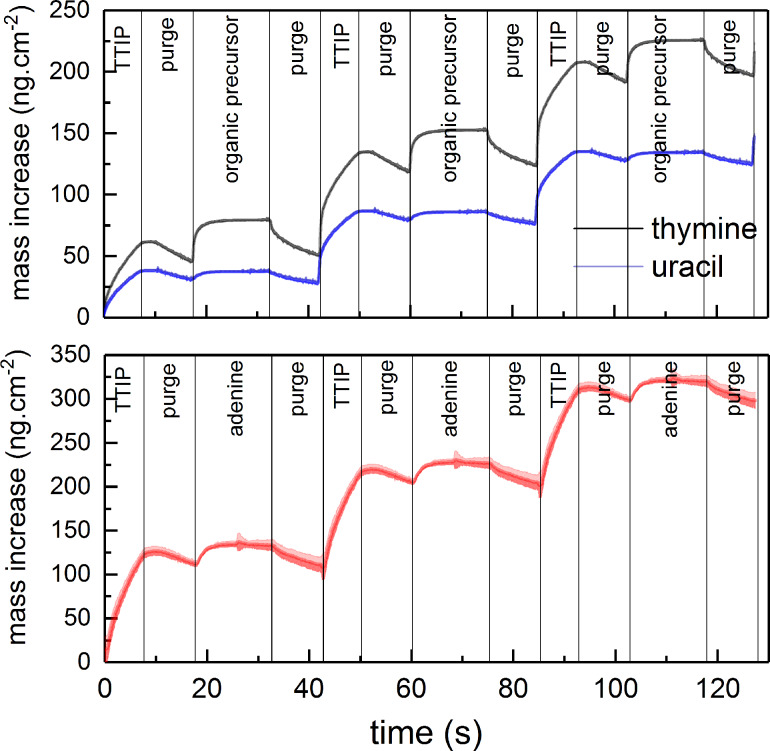
Evaluation of mass increase during growth by QCM using TTIP and thymine (at 225 °C), uracil (at 225 °C) (top graph), or adenine (250 °C) (bottom graph). The shaded area represents the standard deviation during 16 cycles, where the noise in the middle of the adenine pulse stems from edge effects of the statistics.

The growth dynamics of all three systems were investigated using in situ QCM, as shown in Figure 2 and Figure 3 and summarized in Table 1. We used two different approaches for the QCM investigations in these studies. When mapping for suitable pulsing and purging parameters, we applied a basis pulsing scheme of 1 s TTIP, 1 s purge, 2 s organic precursor, 1 s purge and varied one of these parameters while recording the growth rate over 20 consecutive cycles using the middle 16 cycles for statistics, as shown in Figure 2. This approach resembles a practical growth mode where the pulse times are kept sufficiently long for homogeneous growth but shorter than what is required for complete saturation. Long pulse times typically add to the required purge times and lead to impractical process times. A possible bi-reaction during ALD growth is the production of a CVD component leading to increased growth for prolonged pulsing, particularly at higher temperatures [24]. Normally, this contribution is ignored as long as an ALD mode dominates the overall film growth. Such a CVD contribution is limited when a practical growth mode is considered sufficient.

**Table 1:** Deposition conditions for QCM investigation and obtained pulse and purge parameters.

Organic precursor	Sublimation temperature (°C)	Deposition temperature (°C)	TTIP pulse (s)	Purge (s)	Organic precursor pulse (s)	Purge (s)

Thymine	207	225	1	2	1	1
Uracil	207	225	1	2	1	1
Adenine	210	250	1	2	1	1

When highlighting the overall growth dynamics, rather long pulse and purge times for all precursors are used. In this case, a pulse scheme of 7 s TTIP, 10 s purge, 15 s organic precursor, 10 s purge was used and the QCM results were averaged over 16 consecutive cycles (Figure 3). Such an approach highlights possible saturation levels, CVD-components and loss during purging, while attempts to extract practical pulse and purge times may be masked by effects from over-pulsing.

When following the first approach, all systems show a two-step reaction for pulsing of TTIP where more than half of the final mass is reached already within the first 0.25 s for the thymine and uracil case, while 0.5 s is required for the adenine system. The mass continues to increase for prolonged pulse times of TTIP, although at a significantly lower rate. A purge time of 2 s is required after TTIP for the thymine case, indicating that most of the mass increase after the first 0.25 s of TTIP pulse is due to physisorption. Any similar effects of purge time after TTIP for the uracil and adenine system are of much smaller magnitude.

For pulsing of the organic components, a two-step reaction is also observed, although notably slower, indicating a transition around 1 s pulse. Likewise, a purge time of 2 s is required after thymine while similar effects are much smaller for purging of uracil and adenine.

The origin of this is better discussed when inspecting the QCM results from the long-pulse sequences given in Figure 3. It is apparent that the thymine system shows a larger loss of material during pulsing after both TTIP and the organic precursor than any of the other systems. While the mass increase during pulsing of uracil and adenine is hardly notable in comparison to what is observed when thymine is pulsed. However, the overall growth is not determined by what is gained during an individual pulse, but its overall gain during a complete cycle.

In an initial attempt to explain the growth, one can apply ligand exchange reactions between TTIP and functional groups on the organic molecules. In this case the functional groups are protons on the amines or protons on tautomers of the ketones of thymine and uracil. Any such reaction should lead to loss of one isopropanol from TTIP with a gain of one organic molecule. The mass of isopropanol is 60.10 u, while thymine, uracil and adenine is 126.12, 112.08 and 135.13 u, respectively, approximately twice that of isopropanol. Based on the QCM long-pulse sequences, the overall mass gain during pulsing of any of the organic molecules is almost negligible when mass loss during the subsequent purging is included. This observation may indicate that two isopropanol molecules are lost for each organic molecule reacted during its pulse. Such a reaction is possible when considering that all organic molecules have at least two reactive sites. On the other hand, this implies that no exchange reaction takes place during the pulsing of TTIP. Such a scheme corresponds well with the QCM results showing a significant mass increase during TTIP pulsing. To accommodate for such a growth mode, the TTIP must be able to coordinate to the previously reacted nucleobases and form sufficiently thermally stable complexes without release of isopropanol.

**Figure 4:**
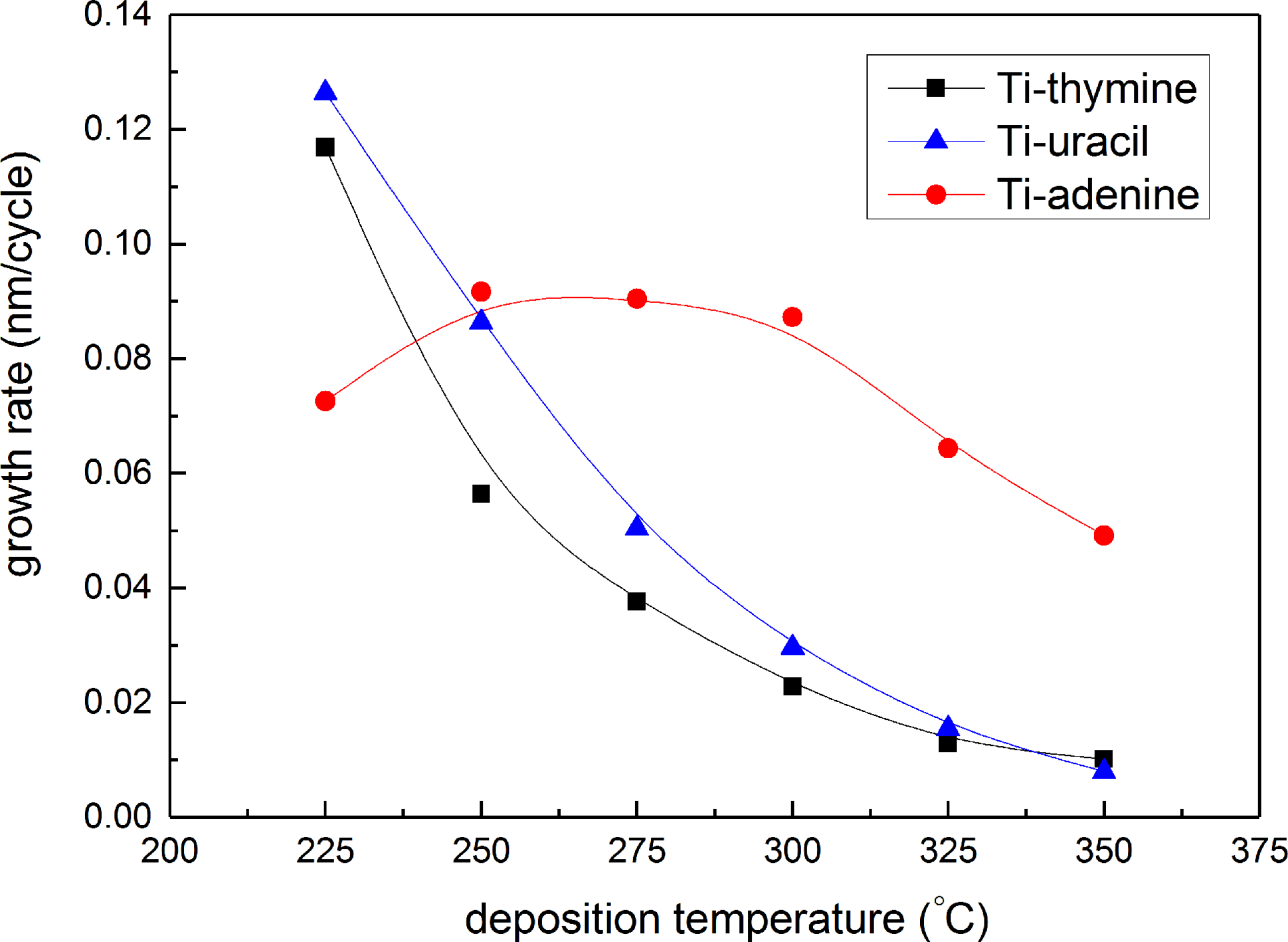
Film growth as a function of deposition temperature for TTIP and thymine (black squares), adenine (red circles), and uracil (blue triangles).

The growth rate of the systems was further investigated as a function of deposition temperature in the range 225–350 °C (Figure 4). The growth rate of the thymine and uracil systems shows a strong dependence with deposition temperature while the adenine system was relatively constant between 250–300 °C, indicating a possible ALD window, before decreasing at higher temperatures.

**Figure 5:**
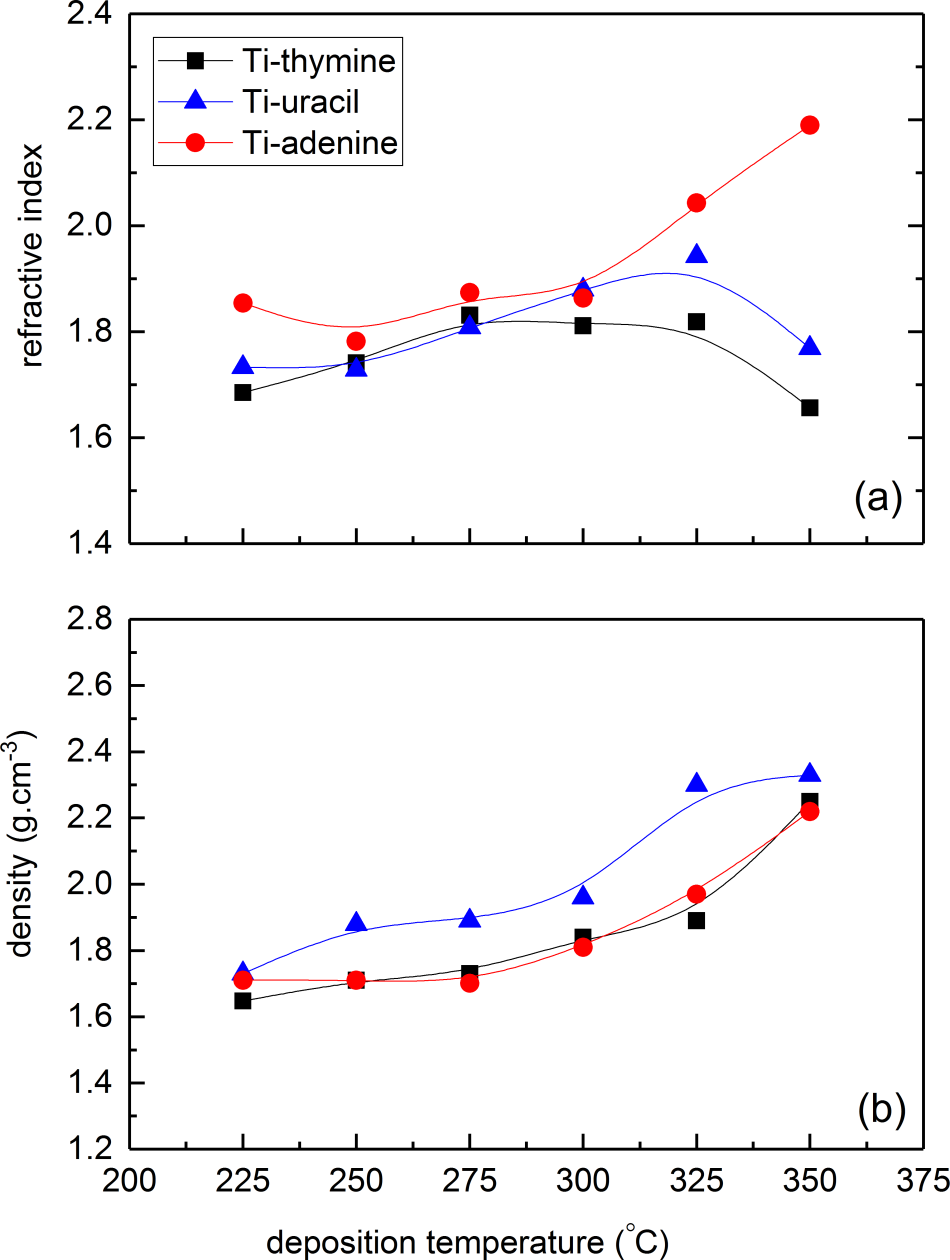
(a) Refractive index at 632.8 nm as measured by spectroscopic ellipsometry and (b) film density as measured by X-ray reflectivity (XRR) as a function of substrate temperature for TTIP and adenine (red circles), thymine (black square) and uracil (blue triangle).

The evolution in refractive index, as measured by ellipsometry, and density, as measured by X-ray reflectivity (XRR), as a function of deposition temperature, is given in Figure 5a and 5b. The index of refraction increased slightly with deposition temperature for all systems. The only discrepancy from this trend was observed for thymine and uracil films deposited at 350 °C. Overall, the refractive index increased from 1.67 to 2.19 over the temperature range of 225 to 350 °C. The density of all the systems increased with deposition temperature from roughly 1.7 g cm^−3^ at 225 °C to 2.3 g cm^−3^ at 350 °C. For comparison, the refractive index and density of anatase TiO_2_ films deposited at 225 °C is 2.36 and 3.78 g cm^−3^, respectively. The increase in refractive index with temperature is similar to what we observed for the Ti-amino acids films in our previous study while the density of these materials was less affected by temperature as compared to the density of the Ti-nucleobase films [22].

After one day of exposure in air, the thickness of the Ti-thymine and Ti-uracil films increased by ≈10 and ≈8%, respectively, while the refractive index decreased from 1.81 to 1.77, and 1.84 to 1.76, respectively. The thickness and refractive index of Ti-thymine and Ti-uracil as-deposited films stabilized after one day. The change in thickness and refractive index indicates that the films are restructured to a less dense structure. However, XRD analysis did not show any sign of crystallinity for both types of as-deposited films (deposited at 225 °C). The thickness and index of refraction for the Ti-adenine system was virtually unaffected by exposure to air.

**Figure 6:**
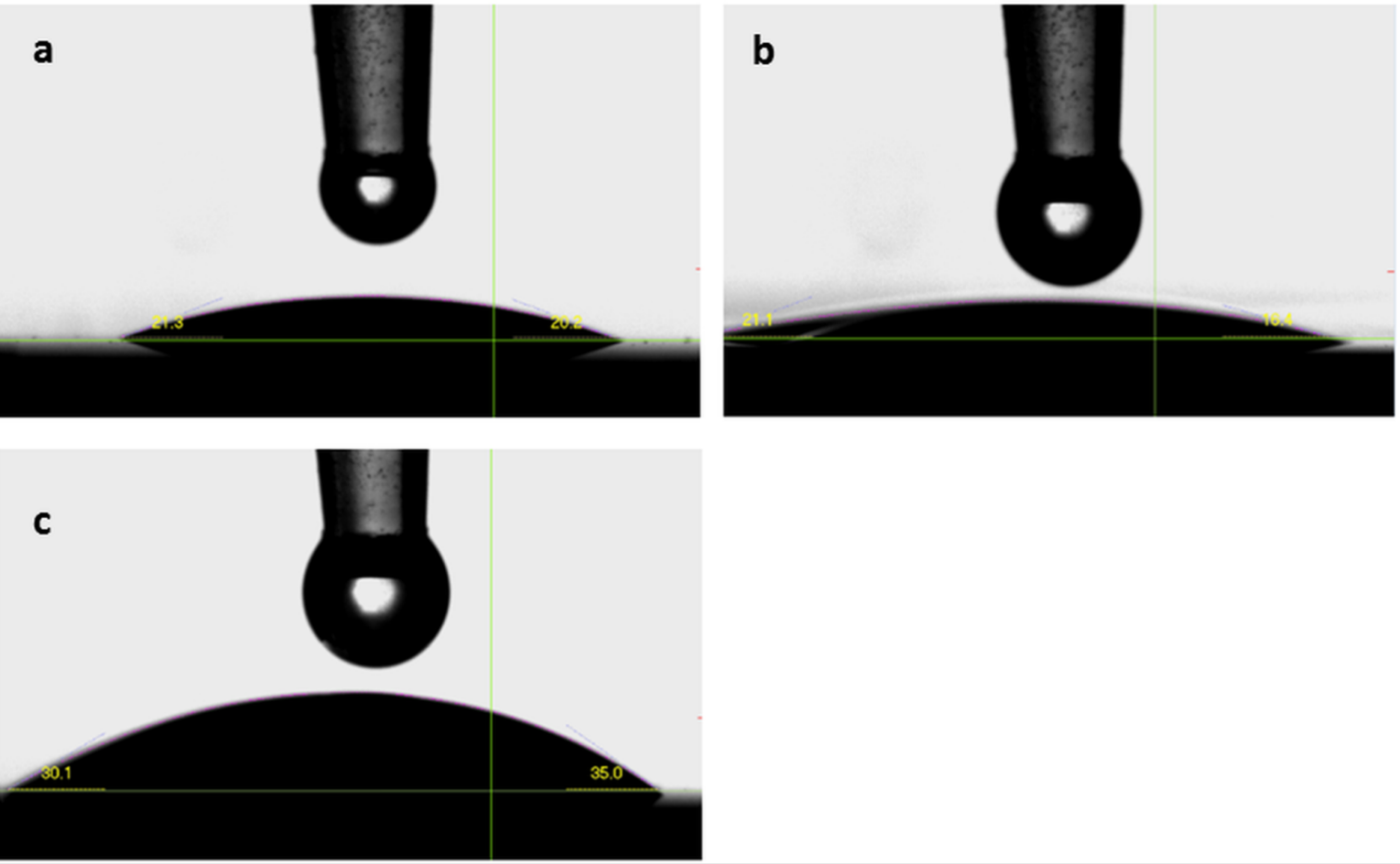
Contact angle between water and films made of TTIP and (a) thymine (b) uracil and (c) adenine.

The wettability of the surfaces was investigated by measuring the contact angle of water for different films using a goniometer. The contact angle was measured on three different spots for each sample. Each spot was measured 10 times in steps of adding 2 µL of water. All three films were relatively hydrophilic (Ti-thymine (deposited at 225 °C) = 19 ± 2°, Ti-uracil (deposited at 225 °C) = 19 ± 1°, and Ti-adenine (deposited at 250 °C) = 45 ± 3°), Figure 6. This hydrophilicity is highly suitable for cell growth purposes. A similar hydrophilic nature was observed for Ti-glycine and Ti-L-aspartic acid films where a contact angle of approximately 30° was measured on the surface of these materials in our previous study [22].

**Figure 7:**
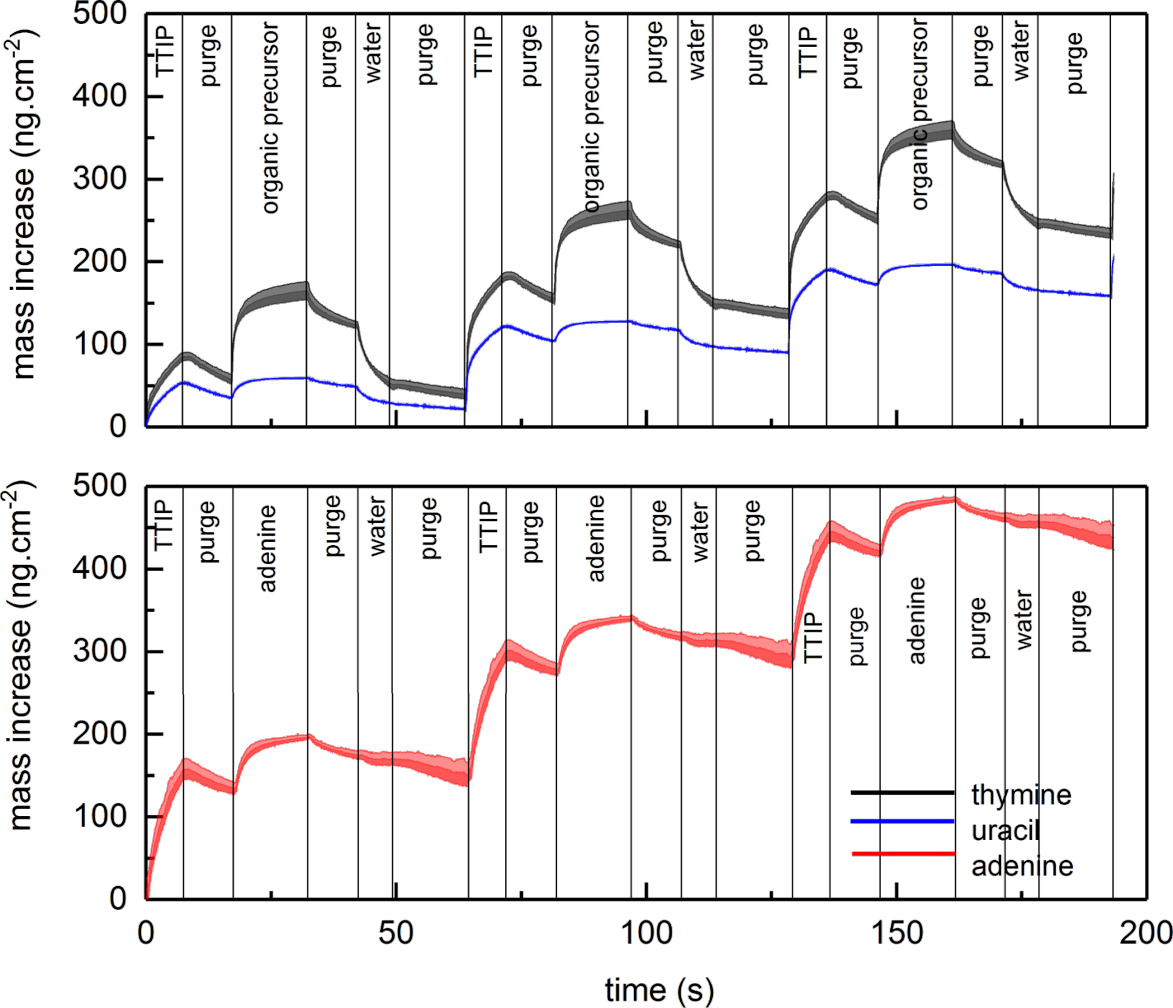
Evaluation of the mass increase during growth measured by QCM using TTIP and thymine (at 225 °C) (top graph), uracil (at 225 °C) (top graph), or adenine (250 °C) (bottom graph), and water. The shaded area represents the statistical variation during 16 cycles.

The growth dynamics of the individual systems were further investigated by adding an extra water pulse after the organic precursor to shed light on the stability of the bonding scheme to the organic building block. The QCM analysis shows a mass decrease during the water pulse followed by a less distinct mass loss during the subsequent purge for the thymine and uracil systems, while the adenine system appears less affected by water (Figure 7). Despite the mass loss during water pulse, the overall growth rates for all systems are increased when water is included, as compared to Figure 3. The QCM measurements were performed at the temperature at which each system had the highest growth rate, 225 °C for thymine and uracil and 250 °C for adenine.

When comparing the growth with and without an additional water pulse after the nucleobase (Figure 3 and Figure 7), the adenine system shows a notably higher stability towards water exposure than for thymine and uracil. For the latter two systems, a notable reduction in mass is observed for the water exposure, indicating that the film is converted towards TiO_2_ and either excess TIP-ligands and/or nucleobases is lost from the film. All of these observations point towards the fact that the adenine system produces a relatively stable complex containing both TIP ligands and adenine. This complex is sufficiently stable towards gaseous water during growth. However, neither of the systems are sufficiently stable towards liquid water.

To further study the effect of water on the film properties, films deposited without the additional water pulse were immersed in water for 15 minutes, three hours and four days. The thickness of the same samples was measured after each period of water treatment. The three hour and four day time periods are the durations in which cells were cultured on these substrates for cell attachment and cell proliferation assays, respectively, in our previous study [23]. Unlike for the Ti-amino acids films that were virtually unchanged by water treatment, the thickness of the films decreases significantly after 15 minutes of water treatment for all systems but thereafter remains almost constant at ≈30 nm, where the data is shown in Table 2.

**Table 2:** Film thickness (nm) after 15 minutes, 3 hours and 4 days of exposure to water as measured by spectroscopic ellipsometry for Ti-thymine (at 225 °C), Ti-uracil (at 225 °C), Ti-adenine (at 250 °C).

Thickness (nm)	Ti-thymine	Ti-uracil	Ti-adenine

Initial thickness	97.7	76.2	87.6
15 minutes	30.6	32.1	31.3
3 hours	29.4	28.5	29.8
4 days	30.9	28.6	30.0

The effect of water treatment on the refractive index and density of the systems were measured after 15 minutes of water treatment (Table 3), indicating some variations in index of refraction, but relatively small variations in film density between the different systems.

**Table 3:** Refractive index at 632.8 nm as measured by spectroscopic ellipsometry and film density as measured by XRR before and after exposure to water for Ti-thymine (at 250 °C), Ti-uracil (at 225 °C), Ti-adenine (at 250 °C).

	density (g cm^−3^)		refractive index	
	before water exposure	after water exposure	before water exposure	after water exposure

Ti-thymine	1.59	2.12	1.73	1.86
Ti-uracil	1.78	2.15	1.76	1.90
Ti-adenine	1.71	2.08	1.80	1.79

**Figure 8:**
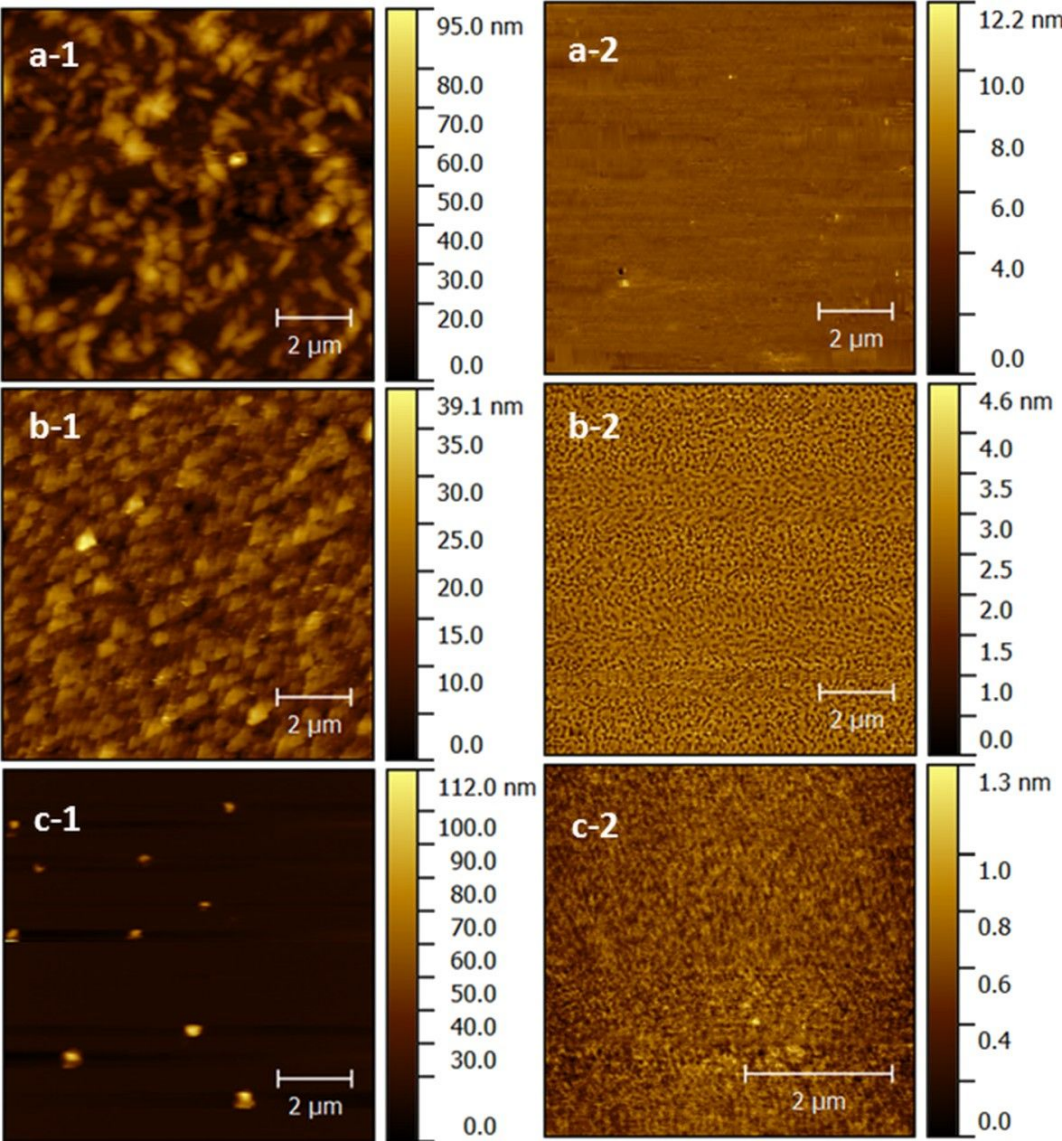
Surface topography as measured by AFM for films deposited using TTIP and (a-1) thymine (at 250 °C, 41 nm), (b-1) uracil (at 225 °C, 56 nm), (c-1) adenine (at 250 °C, 80 nm) and after water treatment (a-2) Ti-thymine (at 250 °C, 15 nm), (b-2) Ti-uracil (at 225 °C, 19 nm), (c-2) Ti-adenine (at 250 °C, 31 nm).

**Figure 9:**
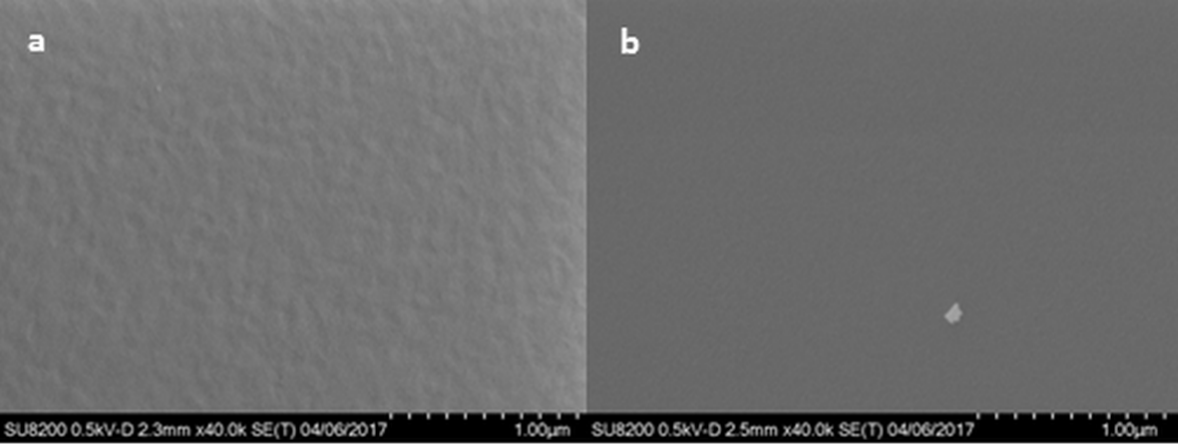
SEM images of (a) the Ti-uracil film deposited (at 225 °C, 19 nm) and (b) the Ti-thymine (at 250 °C, 15 nm) after exposure to water; scale bar 1 µm.

**Figure 10:**
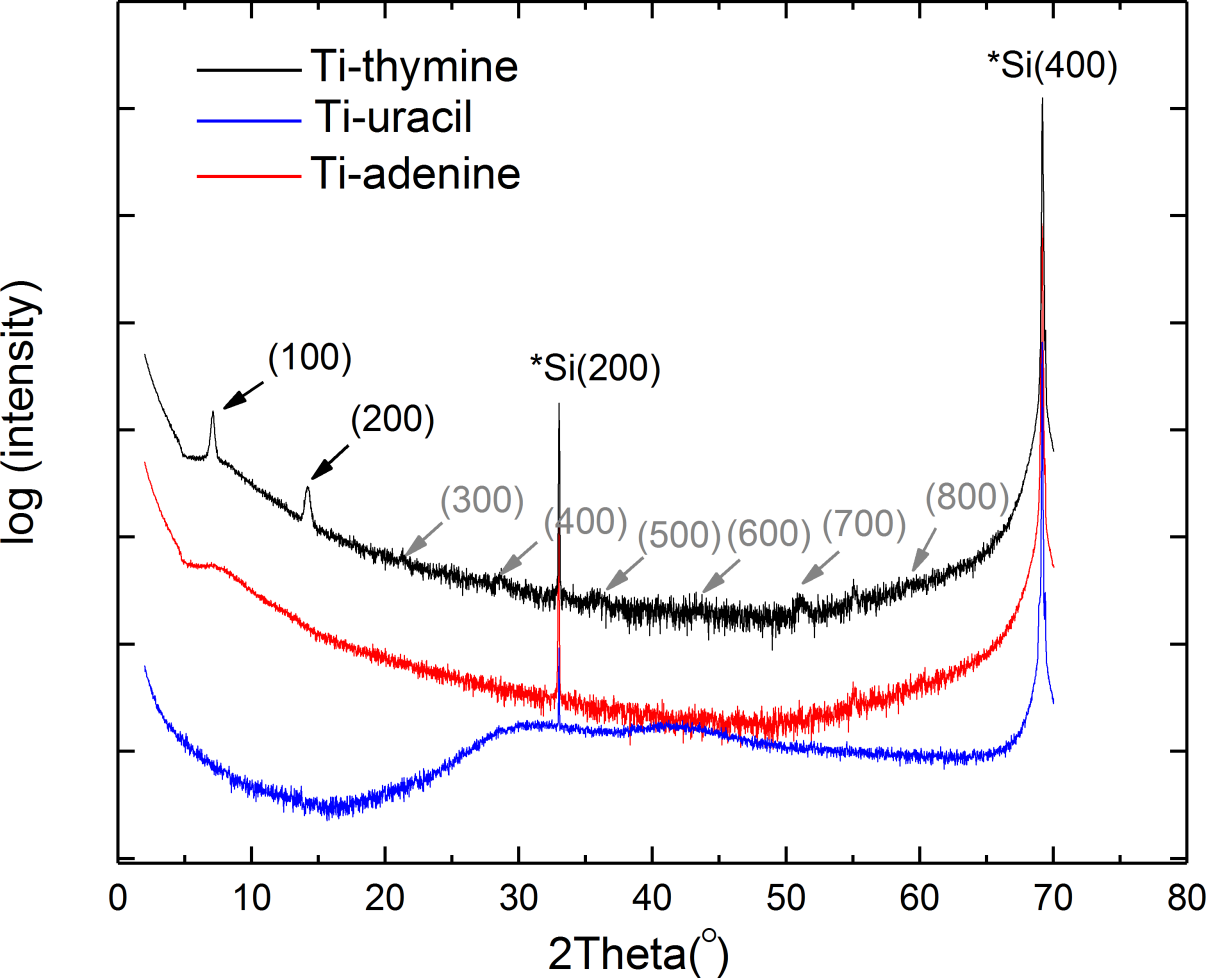
θ–2θ X-ray diffractogram for the Ti-thymine film deposited at 250 °C (black line), Ti-uracil deposited at 225 °C (blue line), and Ti-adenine 250 °C (red line). The reflections for the Ti-thymine film and their multiples are indicated by arrows.

The surface topography of films as-deposited on Si(100) and after being immersed in water for 15 minutes was measured by atomic force microscopy (AFM) (Figure 8). All as-deposited films exhibit high surface roughness; however, the roughness of the Ti-adenine film is caused by small islands appearing on an otherwise almost flat surface. After water treatment, the surface roughness decreases drastically for all three systems and leaves an almost flat surface, except for the Ti-uracil system, where holes with a distinct pattern were observed. This system was also studied more closely with SEM after water treatment (Figure 9a). This SEM image also shows a low surface roughness; however, there is no clear visible identifications of any holes, but rather shallow dimples. For comparison, an SEM image of the Ti-thymine film after exposure to water is included (Figure 9b). The Ti-thymine film deposited at 250 °C shows surface features resembling a crystalline material (Figure 8a) and was further investigated by XRD. While the Ti-thymine film deposited at 225 °C did not show any sign of crystallinity by XRD, the Ti-thymine film deposited at 250 °C showed two distinct XRD reflections at 2θ below 15 degrees (with *d* values of 12.44 Å and 6.22 Å). These are clear multiples of each other and possibly indicate a crystalline layered structure (Figure 10). There are also signs of reflections at higher multiples, but their intensity is rather weak (marked with grey arrow). We were not able to identify any additional reflections and we were also unable to match those found with any known materials containing thymine or titanium oxide. Overall, a more in-depth investigation of the structure of the Ti-thymine films should be performed. No sign of crystallinity was observed for Ti-adenine and Ti-uracil by AFM or XRD.

**Figure 11:**
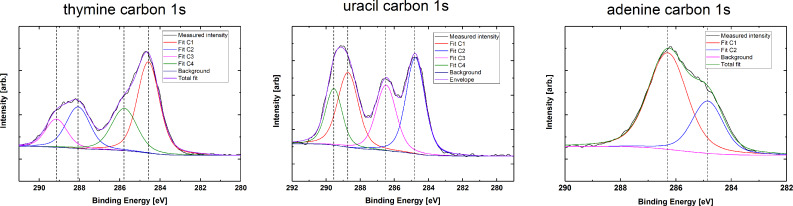
Carbon 1s for films based on adenine, thymine and uracil.

We used XPS as a qualitative investigation of the chemical state in the thin films prior to and after water exposure. The carbon peak of the as-deposited films was used to confirm that the structure of the bases was maintained during deposition. Carbon peak splitting in all three bases is similar to the previously reported XPS results on pure bases in powder form (Figure 11) [25–26].

**Figure 12:**
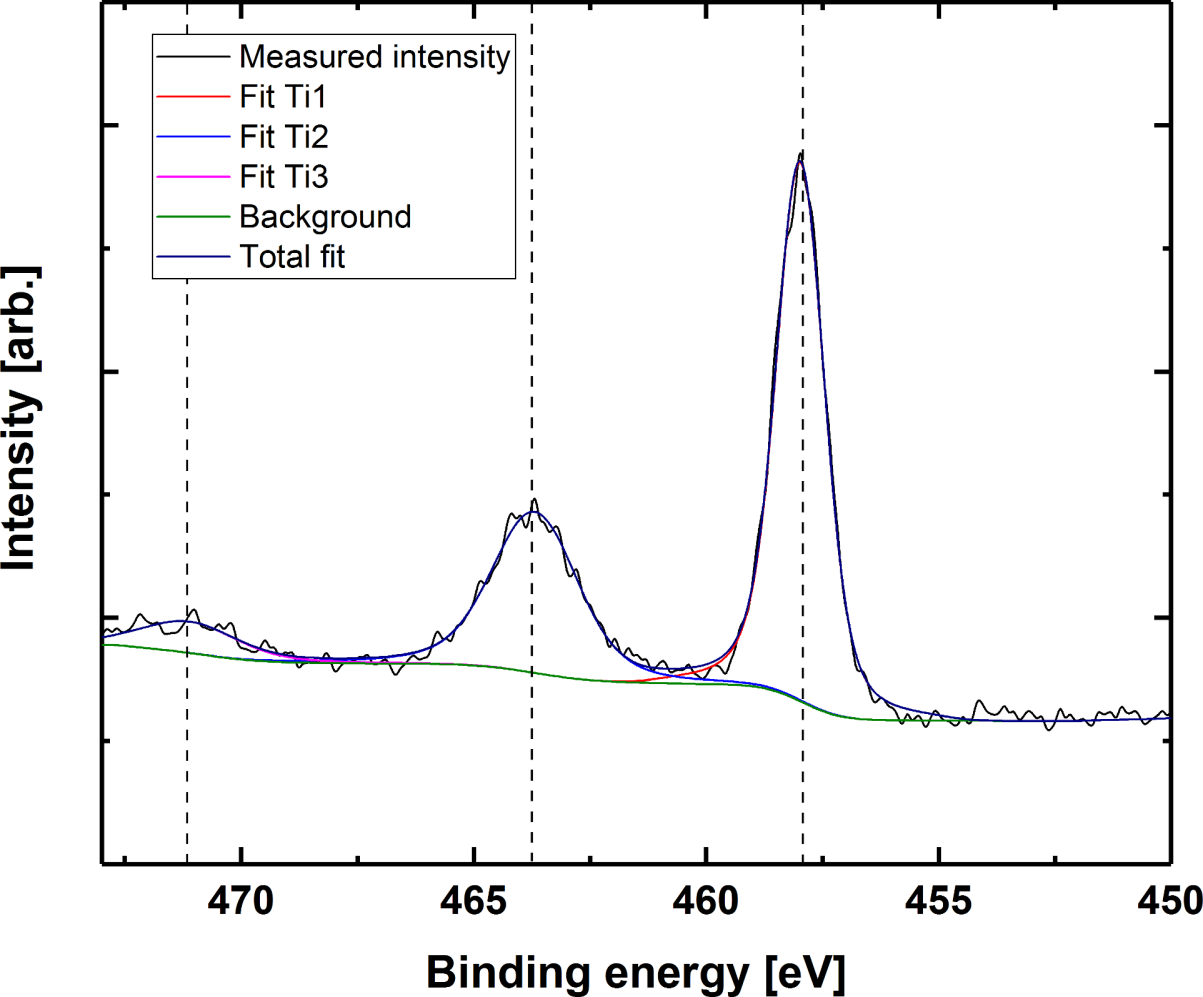
Titanium 2p peak for a hybrid film based on thymine. The binding energy is corrected by calibrating with respect to adventitious carbon (284.8 eV).

The Ti 2p core level spectra are the same for all three sample types, here exemplified by thymine Ti 2p (Figure 12). The 457.8 eV binding energy of the Ti 2p_3/2_ peak point towards predominant Ti–O-type bonding. We confirmed this by the 5.8 eV split spin-orbit energy difference. In addition, we investigated the charge transfer shake-up satellite modes corresponding to the O 2p_eg_ → Ti 3d_eg_ transition [27]. No evidence of pure Ti–N bonding could be observed directly from the Ti peaks, indicating that titanium is not found only coordinated to nitrogen. We cannot, however, rule out the possibility of Ti–N bonding by this observation alone.

**Figure 13:**
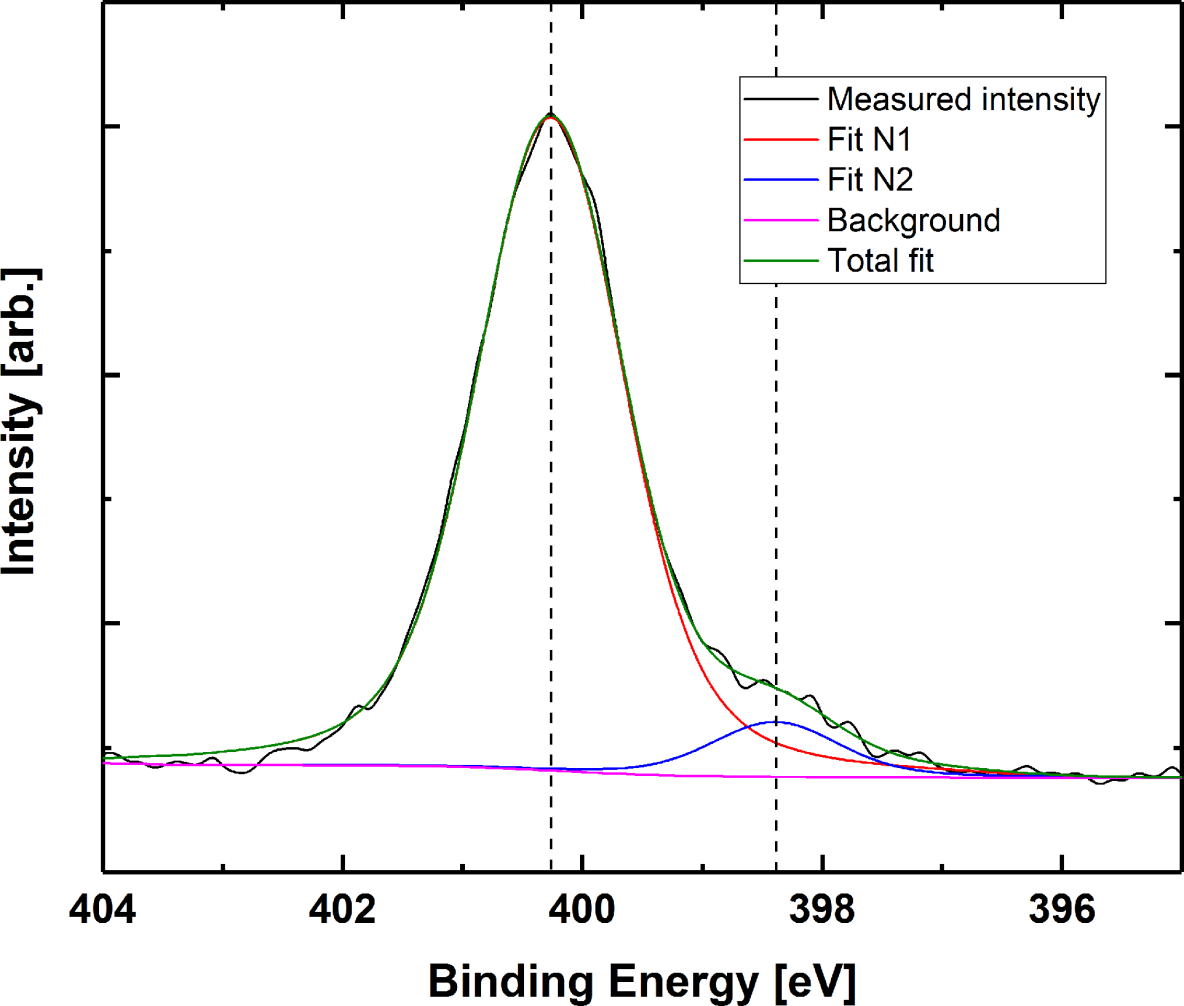
Nitrogen 1s peak for film based on thymine. The binding energy is corrected by calibrating towards adventitious carbon (284.8 eV).

To determine the possible Ti–N coordination, we investigated the N 1s peak for all three bases. The N 1s peaks are slightly asymmetric and require two components for a good fit (see Supporting Information File 1, Figure S1 for a collection of single element peak spectra for all samples before and after water treatment). This points towards at least some degree of Ti–N coordination, as N in the bases should only give rise to one nitrogen component (exemplified for thymine in Figure 13). This second component has an energy corresponding well with the reported energies of O–Ti–N-type bonding [28].

**Figure 14:**
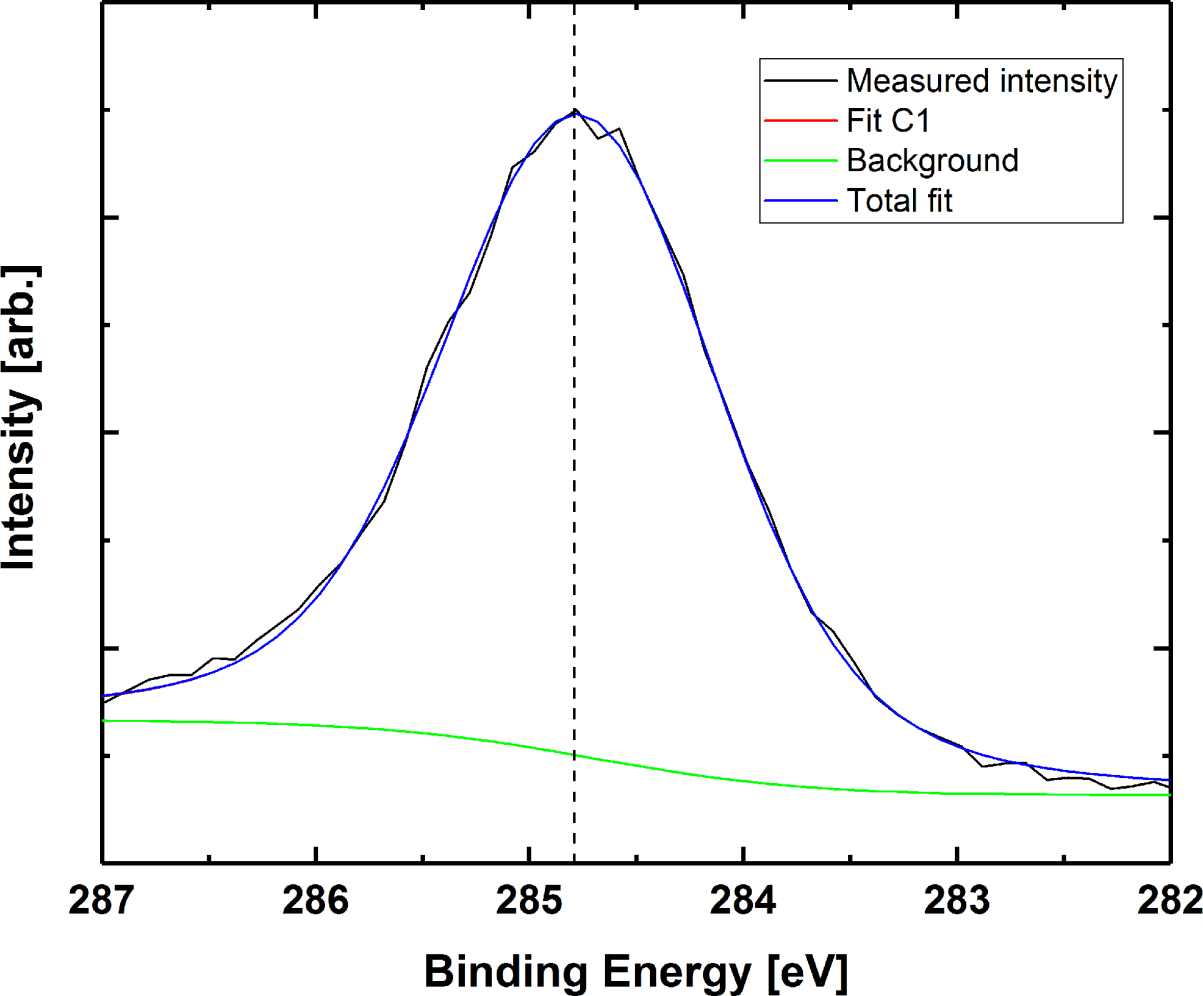
Carbon 1s peak for film based on thymine after water treatment. The binding energy is corrected by calibrating with respect to adventitious carbon (284.8 eV).

Upon water exposure, titanium in the as-deposited films is observed only as TiO_2_. This is evident from the 458.5 eV binding energy for the 2p_3/2_ peak, in addition to the 5.7 eV spin-orbit splitting. Furthermore, the characteristic TiO_2_ XPS satellite peak is observed at 472 eV. A distinct satellite peak is also observed at approximately 472 eV, which is very characteristic for TiO_2_. It should be noted that the Ti signal from a Ti–O–C bond would be indistinguishable from that of pure TiO_2_, so we cannot rule out that some hybrid character is still maintained. Moreover, no trace of the base structure remains. The carbon and nitrogen signals from the bases have much lower intensity, and the residual carbon is attributed to adventitious carbon only. This is exemplified by the C 1s peak of the film based on thymine in Figure 14. This indicates dissolution or decomposition of the bases upon water exposure while TiO_2_ remains in the films. We have also attempted to calculate the elemental composition of the films probed by XPS, although including the remains of adventitious carbon. The results are presented in full in Supporting Information File 1, Table S1, and support that the as-deposited films mainly consist of titanium and their respective bases, while these bases are more or less fully leached out during immersion in water leaving a TiO_2_ surface.

**Figure 15:**
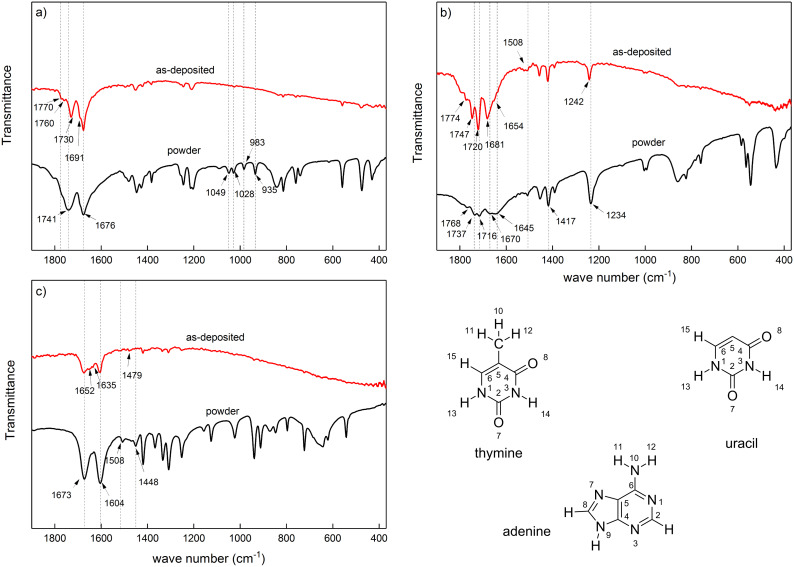
FTIR spectra of powder form and hybrid films of TTIP and (a) thymine (225 °C, 114 nm) (b) uracil (225 °C, 122 nm) and (c) adenine (225 °C, 69 nm).

The FTIR analysis of the hybrid films provides information about the presence of the organic moieties and also the resulting bonding modes between metal and organic molecules. Determining the bonding modes for nucleobases with FTIR is not as straight forwards as for amino acids (as can be seen in [22]); however, comparing characteristic bands of nucleobases before and after coordination with the metal atom can provide information about binding sites in the organic moieties. For thymine, two very strong bands at 1741 and 1676 cm^−1^ are observed in the FTIR spectra of Figure 15a. The lower wavenumber has been assigned to both the stretching vibration of C=C and C4=O, and the vibrational band at 1741 cm^−1^ corresponds to the vibration of C2=O. While both as-deposited Ti-thymine and thymine powder show an absorption band at 1676 cm^−1^, the C2=O vibration for Ti-thymine appears at a lower wavenumber than for the thymine powder (at 1730 cm^−1^). The FTIR spectra for the as-deposited Ti-thymine shows a number of extra vibrational bands compared to thymine powder [29]. In the as-deposited Ti-thymine spectra, two shoulder bands near the vibration of C2=O (at 1770 and 1760 cm^−1^) and one in the vicinity of the C=C and C4=O vibration band (shoulder band at 1691 cm^−1^) were observed. This can be an indication of a change in surrounding environment of thymine molecules and especially oxygen from the carbonyl groups after coordination with titanium. The frequencies (cm^−1^) and assignment of other observed vibration bands for Ti-thymine and thymine powder are in accordance with previous studies on IR spectra of thymine [29].

The region 1600–1800 cm^−1^ is the double bond stretching region for uracil, and the bands belonging to C5=C6, C2=O and C4=O are expected to be in this region (Figure 15b). For uracil powder, two strong stretching modes, C2=O and C4=O, are observed at 1716 and 1670, respectively. Upon coordination with titanium, these two bands appear at 1720 and 1681 cm^−1^, which could indicate that the oxygen in the carbonyl groups are involved in coordination with titanium [30]. Another strong peak at 1737 cm^−1^ followed by a weak peak at 1768 cm^−1^ is observed for the powder, which is due to the Fermi resonance between the overtones and/or combination bands of C=O out-of-plane deformation vibration with C=O stretching vibration. These Fermi resonance vibrations for Ti-uracil are observed at 1747 and 1774 cm^−1^. The carbon double bond vibration is observed at 1645 and 1654 cm^−1^ for uracil and Ti-uracil, respectively [31]. Uracil and adenine have recently been grown with monovalent sodium, divalent alkaline earth (Ba) and trivalent lanthanide (La) metals, and are presented including a thorough FTIR analysis [30,32]. We were not able to detect a similar significant shift in the frequency of N3–H (at 1417 cm^−1^) and N1–H (1508 cm^−1^) after coordination with titanium, as reported previously in Na-uracil thin films and explained by N–H···O hydrogen bond pairs formed between uracil molecules [30]. This may be due to the amorphous nature of our Ti-based complex, hampering the arrangement of the nucleobases. The frequencies (cm^−1^) and assignment of other observed vibrational bands for uracil and Ti-uracil are in accordance with previous reports on the IR spectra of uracil [33–34].

Figure 15c shows that adenine has two strong bands at 1673 and 1604 cm^−1^. The band at 1673 cm^−1^ corresponds to the scissoring vibration of NH_2_. Both adenine powder and the as-deposited Ti-adenine film show this band at 1673 cm^−1^. Upon coordination with titanium, a shoulder band appears near the characteristic peak of the scissoring vibration of NH_2_ at 1652 cm^−1^ in Ti-adenine spectra, which suggests that the amine group is involved in bonding with titanium. The skeletal stretching vibration is expected to appear in the 1600–1450 cm^−1^ region. The strong band at 1604 cm^−1^ and the weak band at 1635 cm^−1^ arise from in-plane vibration of the six-membered ring in adenine. The band at 1508 cm^−1^ shifts to higher frequencies (1515 and 1523 cm^−1^) in the case of Ti-adenine, which indicates a change in vibration of the six-membered ring, after coordination with titanium occurs. The band at 1448 cm^−1^, assigned to the vibration of N9–H, shifts to 1479 cm^−1^ possibly due to binding with titanium through N9 [35–36]. These observations suggest a change in the environment of NH_2_ and N9–H after coordination with titanium.

FTIR analysis was also performed on Ti-nucleobase films prepared with an extra water pulse after the organic molecule, to investigate the hybrid properties of these films. FTIR analysis shows that none of the as-deposited films were completely converted to TiO_2_ by the water pulse. However, a distinct change in characteristic absorption peak of the different organic molecules was observed. The shift in the major characteristic peaks of the organic molecules indicates that water molecules strongly affect the way organic molecules coordinate with titanium (Supporting Information File 1, Figure S2). The QCM result implies that the complex between titanium and adenine is the strongest among the three nucleobases studied here. To investigate this further, a reaction between TTIP and adenine was designed in the bulk phase and FTIR was used to analyze the final product. This experiment was designed to investigate if a stable complex can be formed in the reaction between TTIP and adenine. In this experiment, 0.025 mol adenine was mixed with an excess amount of TTIP (0.1 mol) and stirred under argon atmosphere for 1 hour at 50 °C. The isopropanol was later removed under vacuum for two days at 70 °C and the resultant compound was analyzed with FTIR and compared with adenine pressed into a KBr pellet. Although we were not able to identify the exact final product with FTIR, the results point towards formation of a complex between adenine and titanium that causes a shift in the characteristic peaks of adenine (Supporting Information File 1, Figure S3).

## Discussion

An ALD-type growth is observed for all three TTIP/nucleobase systems (Figure 2 and Figure 3). Judging from the combined results, the overall growth is probably not according to a simple ligand exchange mechanism between isopropanol and available protons on the nucleobases. The presence of the nucleobases in the final product is proven for all systems by FTIR as well as XPS. The FTIR analysis confirms that the carbonyl groups in thymine and uracil are active in bonding, while the primary amine group in adenine takes part. Neither of the systems shows dominant Ti–N bonding schemes, but rather Ti–O bonds. This is particularly informative for the adenine system as it indicates that a significant portion of the TIP ligands are intact in the film during growth (the adenine molecule does not contain any oxygen). XPS analysis shows that the oxygen peak of adenine has split (data not shown). The main peak is consistent with TiO_2_, while the second peak has an energy corresponding well with Ti–O–C. This could indicate the presence of unreacted TIP ligand; however, this peak could also originate from C=O bonds from the surface. After exposure to water, the second peak decreases significantly, which points towards the reaction of TIP ligand with water, although this also could indicate that water removes some of the C=O-bonds from the surface, but this is less probable.

Even though the adenine system shows an apparent ALD window between 250 and 300 °C, all systems result in a reduced growth rate with increased temperature. This should be considered in comparison with the TTIP/water system and its apparent ALD window between 190 and 240 °C [37]. The constant growth rate in the ALD window of the latter case is claimed to result from a balance between hydrolysis and thermal decomposition of TTIP itself, hence an ever increasing growth rate with prolonged pulsing of TTIP above 200 °C [37]. In contrast to our current observations, the TTIP/H_2_O system is reported to continue increasing its growth rate with higher temperatures due to the thermal decomposition of TTIP. The lack of such behavior for the TTIP/nucleobase systems indicates that the decomposition mechanism described in [37] does involve water in a manner that our nucleobases are not able to provide. We observed similar trends to our nucleobases in MLD growth rate of titanium-amino acid systems where the growth rate of glycine and L-aspartic acid systems also declined over a large temperature range [22]. Our observations of the decrease in growth rate can be due to increased thermal motion of adsorbed molecules and thus increased steric hindrance. It can also be due to desorption of chemisorbed precursors. However, the fact that the pattern in growth rate is opposite to the TTIP/H_2_O system reduces the likelihood that thermal decomposition of TTIP plays a major role in the film growth.

The refractive index and the density of the films did not change notably for any of the systems for deposition temperatures up to 300 °C, indicating relatively little variation in the deposited material with temperature. The thickness of the films was significantly reduced while the density of as-deposited films increased slightly after 15 minutes of exposure to water, but remained notably lower than what expected for bulk TiO_2_. It is obvious that the nucleobases leached out from the film, probably leaving a collapsed structure high in TiO_2_ content. The AFM analysis indicates a porous structure (Figure 8b), however, attempts of quantifying this by porosity ellipsometry (data not shown) revealed only limited or insignificant porosity (thymine ≈4%, uracil ≈1% and adenine ≈1%). The complex nature of these films therefore remains to be further investigated.

Judging from the observations that most of the nucleobases leached out during the initial 15 minutes of immersion in water, one may question the bioactivity of these films in comparison to pure TiO_2_. Clearly, these films obtained a lower density, amorphous structure (except for thymine deposited at 250 °C) with porous morphology, when compared to anatase TiO_2_. This is verified by our characterization of density and index of refraction of the films, even after leaching. We have recently compared the bioactivity of these films by growth of goblet cells showing comparable cell adhesion, viability and proliferation as anatase TiO_2_, however, all being significantly better than uncoated cover slips [23].

## Conclusion

Thin films of organic–inorganic hybrid materials were successfully deposited using thymine, uracil, or adenine along with TTIP. The films are hydrophilic, however, the nucleobases do leach when the films are immersed in water. The films are amorphous as-deposited, apart from the thymine system deposited at 250 °C that shows a layered structure. The nucleobases coordinate to titanium through their carbonyl groups for thymine and uracil, and through its primary amine for adenine. However, all films bear signs of inclusion of TTIP in the films during growth. This is particularly noticeable for the adenine system where the complex formed is stable towards ambient air.

## Experimental

The films were deposited in an F120-Sat reactor (ASM Microchemistry Ltd.) using TTIP (Sigma ≥ 97.0%) and thymine (Sigma, ≥ 99%), uracil (Sigma, ≥ 99.0%), adenine (Sigma, ≥ 99%) as precursors. The different precursors together with their names as used in this paper are sketched in Figure 1. Nitrogen was used as a carrier gas supplied at a total rate of 500 cm^3^ min^−1^ from a Schmidelin-Sirocco-5 N_2_ generator with a purity of 99.999% with respect to N_2_ and Ar content. The films were deposited on precleaned single crystal substrates cut from Si(100) wafers.

The growth dynamics were investigated in situ by a quartz crystal microbalance (QCM) using a Maxtek TM400 unit and homemade crystal holders. A change in resonance frequency of the crystal is linearly proportional to the mass of the deposited film, according to the Sauerbrey equation [38], and is hence a valuable tool for following the growth. The QCM data were further processed by averaging 16 consecutive cycles for better statistics. The conversion from frequency to mass per area was done by using internal standards throughout the deposition campaign of a material with known growth rate and density, as measured by X-ray reflectivity (XRR). This procedure calibrated for possible variations in surface area of the QCM crystal due to evolution of texture during growth.

The growth rate was measured as a function of deposition temperature for each inorganic–organic precursor pair in the range 225–350 °C. The thickness and refractive index of the films were measured by a J.A. Woollam alpha-SE spectroscopic ellipsometer at an incident angle of 70°. The films were assumed to be transparent and the data were fitted using a Cauchy model. The density of the films was measured by a Bruker AXS D8 advance film diffractometer equipped with a LynxEye strip detector. The thin film diffractometer had a Göbel mirror and a Ge(220) four bounce monochromater for XRR measurements.

X-ray photoelectron spectroscopy (XPS) was performed using a Thermo Scientific Theta Probe Angle-Resolved XPS system. The energy is charge referenced to adventitious C1s, C–C peak, at 284.8 eV. The instrument is equipped with a standard Al Kα source (*h*ν = 1486.6 eV), and the analysis chamber pressure is on the order of 10^−8^ mbar. Pass energy values of 200 and 50 eV were used for survey spectra and detailed scans, respectively. Ti 2p, C 1s, N 1s and O 1s were captured for all samples.

Fourier transform infrared (FTIR) transmission spectroscopy was performed using a Bruker VERTEX 80 FTIR spectrometer to obtain infrared spectra of the films. The system was equipped with a nitrogen purging system. An uncoated Si(100) substrate was used to collect the background.

Atomic force microscopy (AFM) measurements were performed in contact mode using a Park XE70 device. The data were analyzed using the Gwyddion 2.44 SPM visualization tool. The contact angle measurements were performed using a ramé-hart contact angle goniometer and DROP image analysis program.

## Supporting Information

File 1 Additional figures and tables.
