# Supplementary material for: Biocompatible organic–inorganic hybrid materials based on nucleobases and titanium developed by molecular layer deposition
Source: Beilstein J Nanotechnol. 2019 Feb 8;10:399–411. doi: 10.3762/bjnano.10.39 (PMC6369986; doi:10.3762/bjnano.10.39)
Supplement: File 1 — Additional figures and tables. [file Beilstein_J_Nanotechnol-10-399-s001.pdf]

BEILSTEIN JOURNAL OF NANOTECHNOLOGY

Supporting Information

for

Biocompatible organic–inorganic hybrid materials based on nucleobases and titanium developed by molecular layer deposition

Leva Momtazi, Henrik H. Sønsteby and Ola Nilsen

Beilstein J. Nanotechnol. **2019,** *10,* 399–411. doi:10.3762/bjnano.10.39

Additional figures and tables

License and Terms: This is a supporting information file under the terms of the Creative Commons Attribution License (http://creativecommons.org/licenses/by/4.0). Please note that the reuse, redistribution and reproduction in particular requires that the authors and source are credited.
The license is subject to the *Beilstein Journal of Nanotechnology* terms and conditions: (https://www.beilstein-journals.org/bjnano)

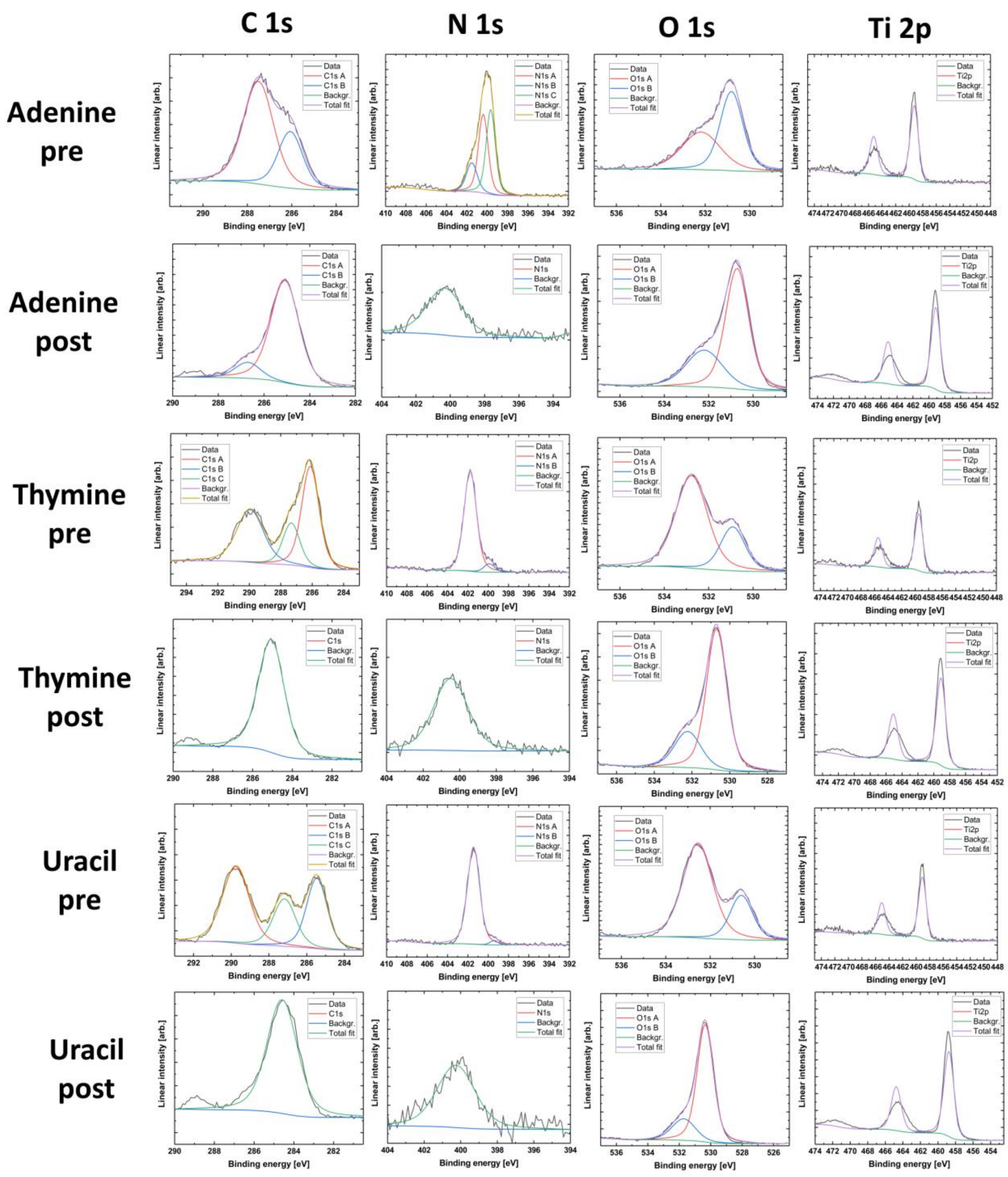

Figure S1: Carbon 1s, nitrogen 1s, oxygen 1s and titanium 2p XPS data with accompanying peak fits (Avantage), for adenine, thymine and uracil hybrid Ti-films prior to and post water treatment.

Table S1: Elemental analysis in the top layer of the hybrid Ti-films of adenine, thymine and uracil prior to and post water treatment, as measured by XPS.

	Adenine pre [%]	Adenine post [%]	Thymine pre [%]	Thymine post [%]	Uracil pre [%]	Uracil post [%]
Carbon (C 1s)	43.1	36.87	49.46	31.02	44.71	29.24
Nitrogen (N 1s)	33.82	2.24	18.50	3.18	20.79	1.50
Oxygen (O 1s)	17.32	46.46	27.95	49.74	30.25	51.45
Titanium (Ti 2p)	5.76	14.43	4.09	16.06	4.25	17.81

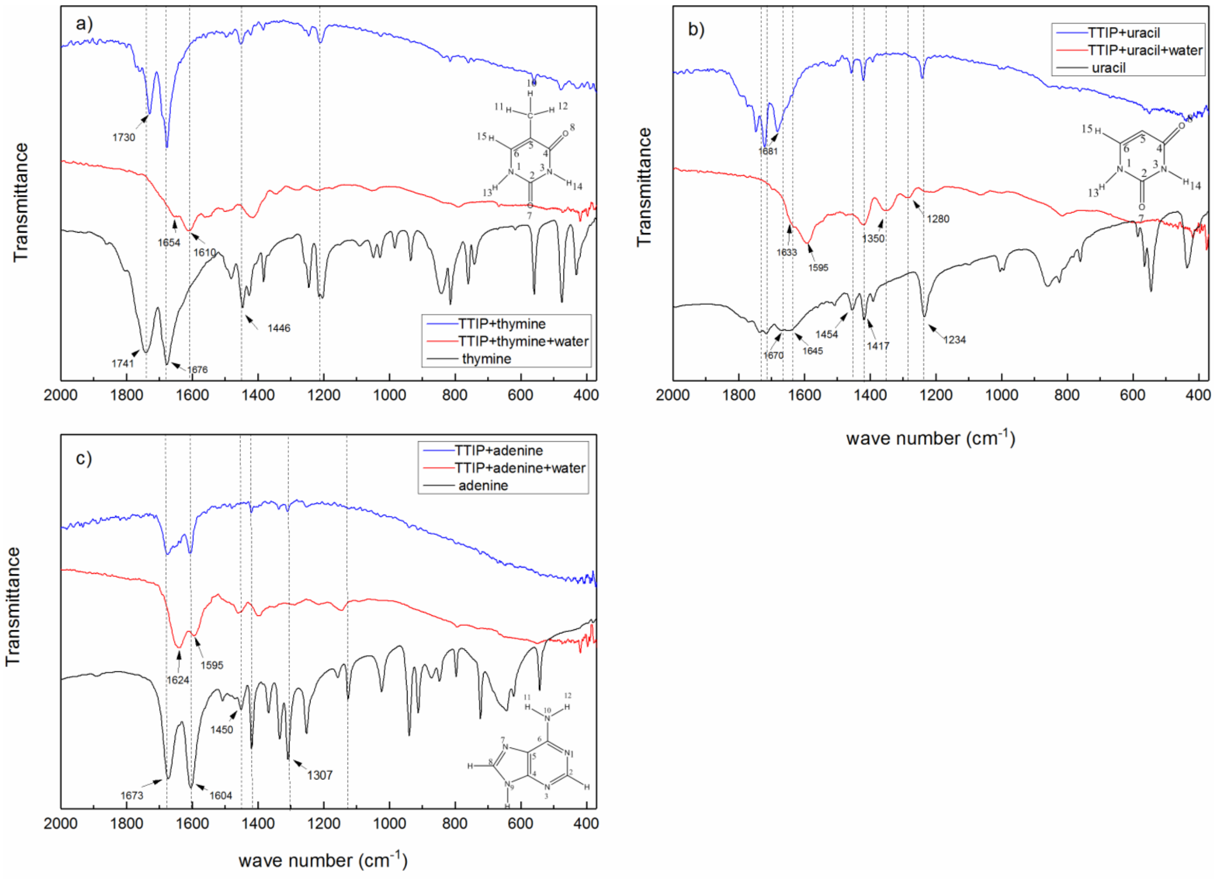

Figure S2: FTIR spectra of (a) thymine powder (black line), hybrid film based on TTIP/p/thymine/p/water/p pulse sequence (red line) and hybrid film based on TTIP/p/thymine/p pulse sequence (blue line) (b) uracil powder (black line), hybrid film based on TTIP/p/uracil/p/water/p pulse sequence (red line) and hybrid film based on TTIP/p/uracil/p pulse sequence (blue line) (c) adenine powder (black line), hybrid film based on TTIP/p/adenine/p/water/p pulse sequence (red line) and hybrid film based on TTIP/p/adenine/p pulse sequence (blue line)

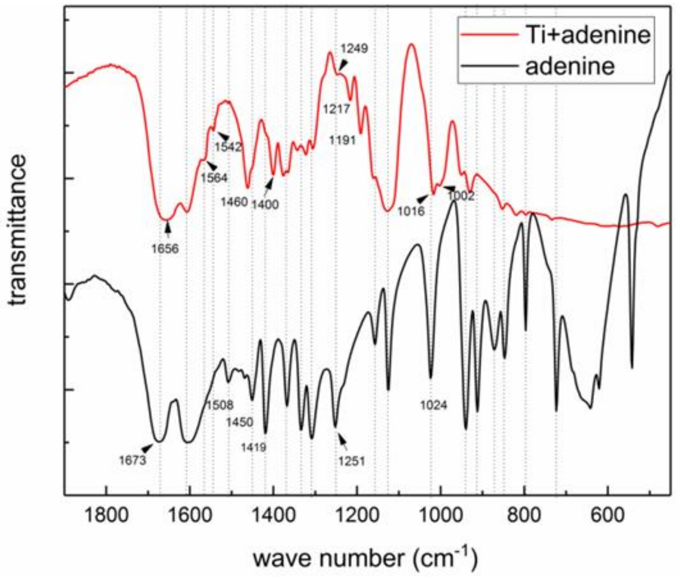

Figure S3: FTIR spectra of adenine pressed in KBr pellet (black line) and Ti-adenine complex (red line).
